# A Systematic Quality Scoring Analysis to Assess Automated Cardiovascular Magnetic Resonance Segmentation Algorithms

**DOI:** 10.3389/fcvm.2021.816985

**Published:** 2022-02-15

**Authors:** Elisa Rauseo, Muhammad Omer, Alborz Amir-Khalili, Alireza Sojoudi, Thu-Thao Le, Stuart Alexander Cook, Derek John Hausenloy, Briana Ang, Desiree-Faye Toh, Jennifer Bryant, Calvin Woon Loong Chin, Jose Miguel Paiva, Kenneth Fung, Jackie Cooper, Mohammed Yunus Khanji, Nay Aung, Steffen Erhard Petersen

**Affiliations:** ^1^NIHR Barts Biomedical Research Centre, William Harvey Research Institute, Queen Mary University, London, United Kingdom; ^2^Barts Heart Centre, St Bartholomew's Hospital, Barts Health NHS Trust, London, United Kingdom; ^3^Circle Cardiovascular Imaging, Calgary, AB, Canada; ^4^National Heart Centre Singapore, Singapore, Singapore; ^5^Cardiovascular and Metabolic Disorders Program, Duke-National University of Singapore, Singapore, Singapore; ^6^Yong Loo Lin School of Medicine, National University Singapore, Singapore, Singapore; ^7^The Hatter Cardiovascular Institute, University College London, London, United Kingdom; ^8^Cardiovascular Research Center, College of Medical and Health Sciences, Asia University, Taichung, Taiwan; ^9^Department of Cardiology, Newham University Hospital, Barts Health NHS Trust, London, United Kingdom; ^10^Health Data Research UK, London, United Kingdom; ^11^Alan Turing Institute, London, United Kingdom

**Keywords:** cardiac magnetic resonance (CMR), cardiac segmentation, machine learning, automated contouring, quality control, assessment

## Abstract

**Background:**

The quantitative measures used to assess the performance of automated methods often do not reflect the clinical acceptability of contouring. A quality-based assessment of automated cardiac magnetic resonance (CMR) segmentation more relevant to clinical practice is therefore needed.

**Objective:**

We propose a new method for assessing the quality of machine learning (ML) outputs. We evaluate the clinical utility of the proposed method as it is employed to systematically analyse the quality of an automated contouring algorithm.

**Methods:**

A dataset of short-axis (SAX) cine CMR images from a clinically heterogeneous population (*n* = 217) were manually contoured by a team of experienced investigators. On the same images we derived automated contours using a ML algorithm. A contour quality scoring application randomly presented manual and automated contours to four blinded clinicians, who were asked to assign a quality score from a predefined rubric. Firstly, we analyzed the distribution of quality scores between the two contouring methods across all clinicians. Secondly, we analyzed the interobserver reliability between the raters. Finally, we examined whether there was a variation in scores based on the type of contour, SAX slice level, and underlying disease.

**Results:**

The overall distribution of scores between the two methods was significantly different, with automated contours scoring better than the manual (OR (95% CI) = 1.17 (1.07–1.28), *p* = 0.001; *n* = 9401). There was substantial scoring agreement between raters for each contouring method independently, albeit it was significantly better for automated segmentation (automated: AC2 = 0.940, 95% CI, 0.937–0.943 vs manual: AC2 = 0.934, 95% CI, 0.931–0.937; *p* = 0.006). Next, the analysis of quality scores based on different factors was performed. Our approach helped identify trends patterns of lower segmentation quality as observed for left ventricle epicardial and basal contours with both methods. Similarly, significant differences in quality between the two methods were also found in dilated cardiomyopathy and hypertension.

**Conclusions:**

Our results confirm the ability of our systematic scoring analysis to determine the clinical acceptability of automated contours. This approach focused on the contours' clinical utility could ultimately improve clinicians' confidence in artificial intelligence and its acceptability in the clinical workflow.

## Introduction

Cardiac magnetic resonance (CMR) is the gold standard non-invasive imaging modality for accurate quantification of cardiac chamber volume, myocardial mass and function (1). Image segmentation is an essential step in deriving such quantitative measures that provide valuable information for early detection and monitorning of a wide range of cardiovascular diseases (CVDs) (2–5). However, manual analysis of CMR images is time-consuming and prone to subjective errors, as the delineation quality strongly depends on the operator's experience (6).

Automated segmentation based on machine learning (ML) algorithms can reduce the inter- and intra-observer variability and speed up the contouring process (7). Additionally, these ML-based methods can expedite the extraction of clinically relevant information from larger image datasets. Although several studies have shown promising results in efficiency and consistency (8–10), important challenges need to be addressed before automatic contouring methods can be robustly and routinely applied in clinical practice.

Automatically generated contours often require manual operator corrections to make the results clinically acceptable (11). An adequate quality-based assessment of the performance of such tools is, therefore, needed. Several quantitative measures have been proposed to assess the quality of automated segmentation against “ground truth” reference, represented by manual contours (12). The most commonly used metrics are based on position, distance and volume overlap (13). However, these measures have a low correlation to clinical contour quality and may not predict clinicians' trust for the contouring method. Therefore, evaluating the performance of automated algorithms in terms of clinical applicability is needed.

In this paper, we describe a new method for assessing the quality of ML outputs by involving clinicians during the algorithm validation process. We evaluate the effectiveness of the proposed method as it is employed to systematically analyse the quality of an automated contouring algorithm.

We used a quality control (QC) scoring system to record the judgments of blinded clinicians on the quality of randomly presented manual and automatic contours. We evaluated the clinical acceptability of automated contouring by analyzing the degree of agreement between two segmentation methods based on the quality scores. We also assessed the scoring system's reliability between the clinicians and whether factors potentially making the segmentation more challenging would have affected the quality judgment.

## Methods

### Dataset

The dataset included in this study contained short-axis (SAX) cine CMR images from 217 subjects who were participants from the National Heart Center Singapore (NHCS) Biobank. Patients' information on sex and age were not available as data were anonymised before the analysis. The study population was heterogeneous and comprised both healthy subjects (*n* = 42) and patients with different CVDs: dilated cardiomyopathy (DCM) (*n* = 33), hypertension (HTN) (*n* = 107), hypertrophic cardiomyopathy (HCM) (*n* = 13), ischaemic heart disease (IHD) (*n* = 15), left ventricular non-compaction (LVNC) (*n* = 6), and myocarditis (*n* = 1). It should be noted that our dataset did not include conditions with uncertain diagnosis (e.g. suspected cardiomyopathy) or with non-pathological changes in cardiac morphology (e.g. athlete's heart). Furthermore, only the images deemed of good quality were included and retained for further analysis.

### Manual Image Analysis

The manual segmentation was performed by a group of experienced investigators from the National Heart Centre Singapore (NHCS), consisting of CMR consultants, well-trained clinical research fellows and engineers, using a standardized protocol described in detail in a separate publication (14). Two operators with over five years' experience (LTT and CWLC) then checked the contouring quality to select the studies deemed for further analysis.

Specifically, left ventricle (LV) endocardial and epicardial borders and the right ventricle (RV) endocardial borders were manually traced in SAX slices at end-diastole (ED) and end-systole (ES) time frames using the cvi42 post-processing software (version 5.1.1, Circle Cardiovascular Imaging Inc., Calgary, Alberta, Canada). ED and ES phases were defined, respectively, as the image with the largest and smallest LV blood volume at visual inspection. The manual contours and the corresponding images were saved for later processing.

### Automated Image Analysis

A ML algorithm trained at Circle Cardiovascular Imaging Inc. was then applied to the same set of manually annotated SAX images to obtain automated contours. A deep convolutional neural network (CNN) was trained to perform SAX image segmentation. A similar model architecture as that of the standard U-Net was adopted for this purpose, along with various data augmentation techniques to enhance the generalizability of the trained model (15). The model was trained on the UK Biobank (UKB) data, a large-scale population-based imaging study mainly composed of healthy subjects as well as individuals with some pathological conditions including HCM and HTN (16, 17). The output of the model was a pixel level classification of the image, where the classes represented one of the SAX tissues including the LV endocardium, LV epicardium, and RV endocardium. The resulting predicted segmentations of the LV and RV were finally saved to be displayed in the contour quality scoring application.

### QC Scoring System

A contour quality scoring application was developed to input clinician's feedback on both manual and automated contours obtained from the same images. The application was composed of a graphical user interface (GUI) to display contoured images and offered user interactivity to rapidly parse through the data and input user's feedback on the quality of the presented contours.

**Figure 1:**
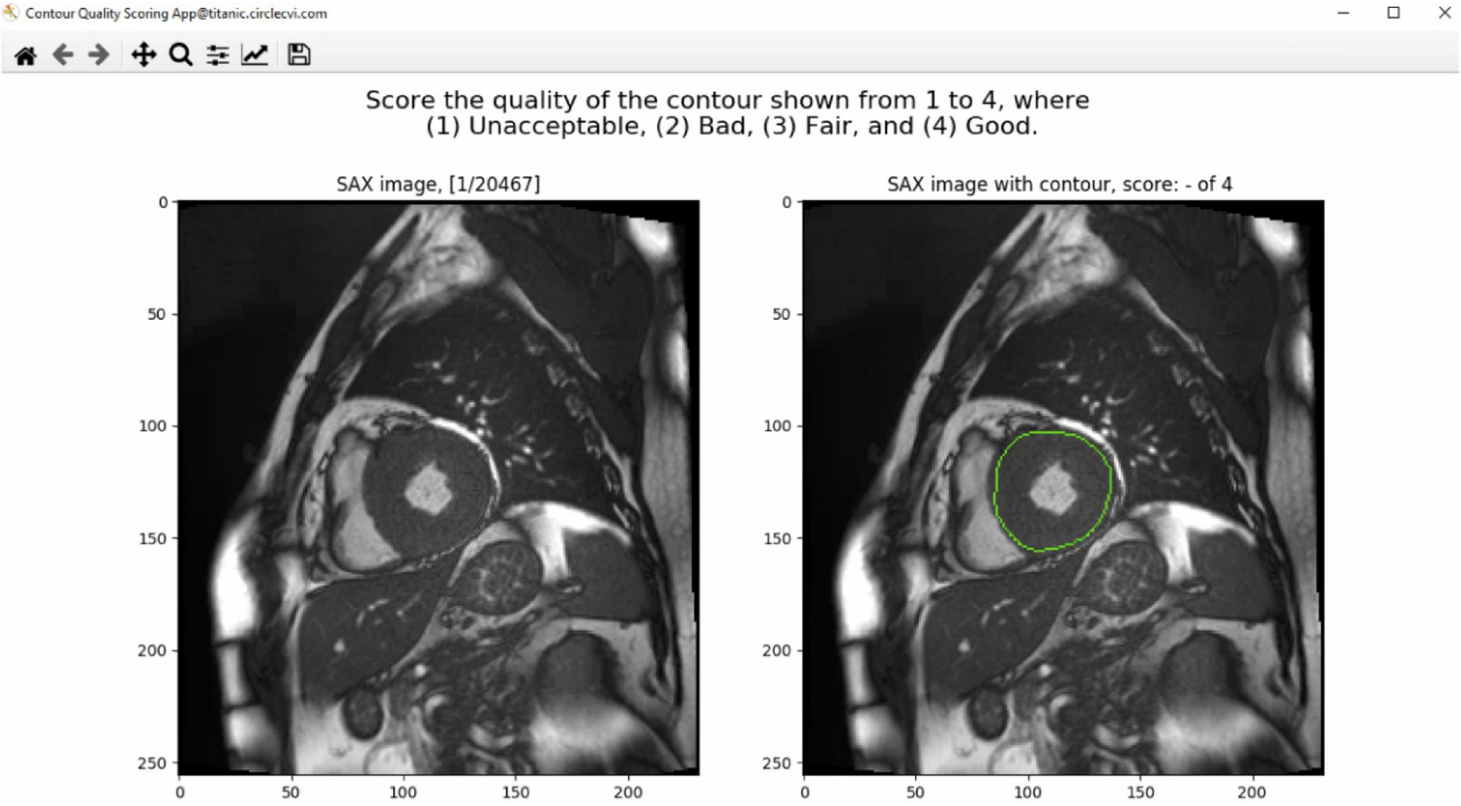
The graphical user interface (GUI) of the contour quality scoring tool. The left panel shows the current SAX image. The right panel shows the same image with overlaid contour to which the rater is asked to assign a quality score. The title above the right panel shows a blank quality score, which will be updated when a value is entered by the user.

The GUI of the contour quality scoring tool is shown in Figure 1. From the dataset constructed with both manual and corresponding automated contours, the application selected a case randomly to present to the user and expected a quality score from a predefined rubric. The left panel of the GUI showed a SAX image with the corresponding index of the image out of the total number of images available in the database. The right panel, instead, showed the same image with an overlaid contour to which the user was asked to assign a quality score. Once the user inputed the score, arrow keys on the keyboard were used to advance to the next available image in the database.

The source of the contour was not shown to the user to avoid causing any unwanted bias in the rating of the contours. In addition, multiple annotations for each image were displayed separately to receive quality rating for each contour type individually. The process continued until the user rated all cases available in the dataset, resulting in ratings received for both source of contours for each image in the dataset.

The UKB SOP for analysis of LV and RV chambers was used as a reference for assessing the quality of the contoured images (18). The quality score was assigned based on how the contour would have affected clinical outcomes or whether it was judged to be clinically plausible or not.

The scoring rubric included scores ranging from one to *four*. A score of one was assigned to significantly inaccurate segmentation and thus considered clinically unacceptable. A score of two was given to bad quality contours, which required significant manual changes to make them clinically acceptable. A score of three was assigned to fair or clinically acceptable contours with minor or negligible inaccuracies in the segmentation considered not clinically relevant. Finally, a score of four was assigned to contours considered of good quality with no changes needed.

**Figure 2:**
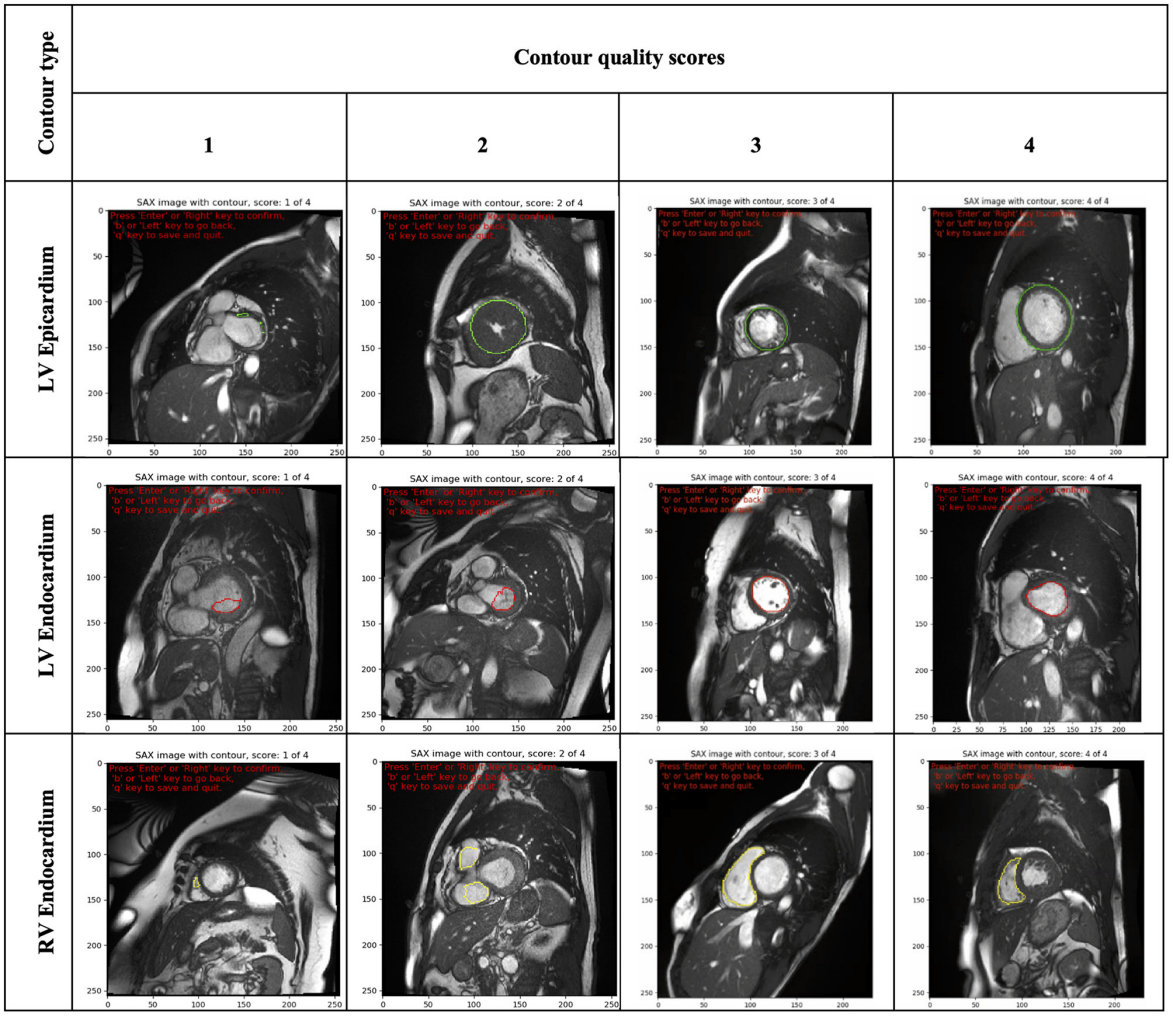
Illustration of some contours (LV epicardial, LV endocardial and RV endocardial) showing the range of quality scores (from 1 to 4). LV, left ventricle; RV, right ventricle.

Furthermore, as images were presented independent of spatiotemporal context, contour quality assessment was mainly based on how well the area of the delineated structure was approximated. Consequently, small contours and small deviations were rarely assigned a quality score of ≤2, as that was considered not clinically relevant. Special attention was given to the RV endocardial contour, especially at SAX basal slices, for which two separate regions may be contoured. In such cases, a score of three was given if the two disjoint contours sufficiently encompassed the underlying anatomy; otherwise, they were scored as two or *one*. An illustration of some contours to which raters assigned different quality scores is shown in Figure 2.

Four clinicians (SEP, MK, KF and ER), from a United Kingdom institution (Barts Heart Center) and varying degrees of experience in analyzing CMR were then asked to independently visualize the contours and assign quality scores using our proposed application. The results were recorded in a database for later analysis.

Firstly, we evaluated the distribution of quality scores between the two segmentation methods across all clinicians. Secondly, we analyzed the quality scores assigned by each rater to evaluate the interobserver variability among the four clincians. Finally, we examined whether there was a variation in quality according to the type of contour, SAX slice level and underlying disease.

### Statistics

Wilcoxon signed-rank test was used to evaluate the differences in the distribution of quality scores between automated and manual for each physician. Combined results over all physicians, and comparison of contour type, slice level and underlying disease were tested using multi level mixed effects ordered logistic regression fitted using the meologit command in Stata. This model takes account of the clustering of ratings from different clinicians for each image. The image was fitted as a random effect with rater and segmentation method fitted as fixed effects. Odds ratios (OR) were obtained from the above model to assess whether the odds of obtaining a higher quality score differed by the method. Interaction terms were fitted to test whether fixed effects differed between manual and automated scores. A *p*-value lower than 0.05 was considered statistically significant.

Interobserver reliability was assessed using Gwet's second-order agreement coefficient with ordinal weighting applied (AC2) (19). Details of the calculation are given in the supplementary methods and the ordinal weights used are shown in Supplementary Table 1. We chose this statistics method over Cohen's Kappa because it has the advantage to assess reliability between multiple observers, and it can be adjusted for both chance agreement and misclassification errors (19, 20). It has been shown to provide a more stable reliability coefficient than Kappa when prevalences differ between the categories as is the case in our data where images were much more likely to be assigned a quality score of four than a score of *one*. The reliability coefficient value and 95% confidence interval (CI) was calculated for both manual and automated contour quality scores. The interpretation of the AC2 coefficient was according to the probabilistic method for benchmarking suggested by Gwet (21). Substantial reliability corresponding to 0.61–0.80 interval was defined acceptable in this study and the benchmark category was selected as the first category with cumulative membership probability exceeding 95%.

Statistical analysis was performed using Python Version 3.6.4 (Python Software Foundation, Delaware USA) and Stata version 17 (StataCorp, Texas).

## Results

Four clinicians with different level of expertise generated a total of 38,991 quality scores. The overall mean quality scores assigned for manual and automated contours were 3.78 ± 0.45 (*n* = 18,516) and 3.78 ± 0.50 (*n* = 20,475), respectively. However the distribution of quality scores differed between the two segmentation methods and was statistically significant (OR (95% CI) = 1.17 (1.07–1.28), *p* = 0.001; *n* = 9401), with automated scoring better than manual on 1,068 occasions compared to 881 occasions where the manual scoring was higher.

**Figure 3:**
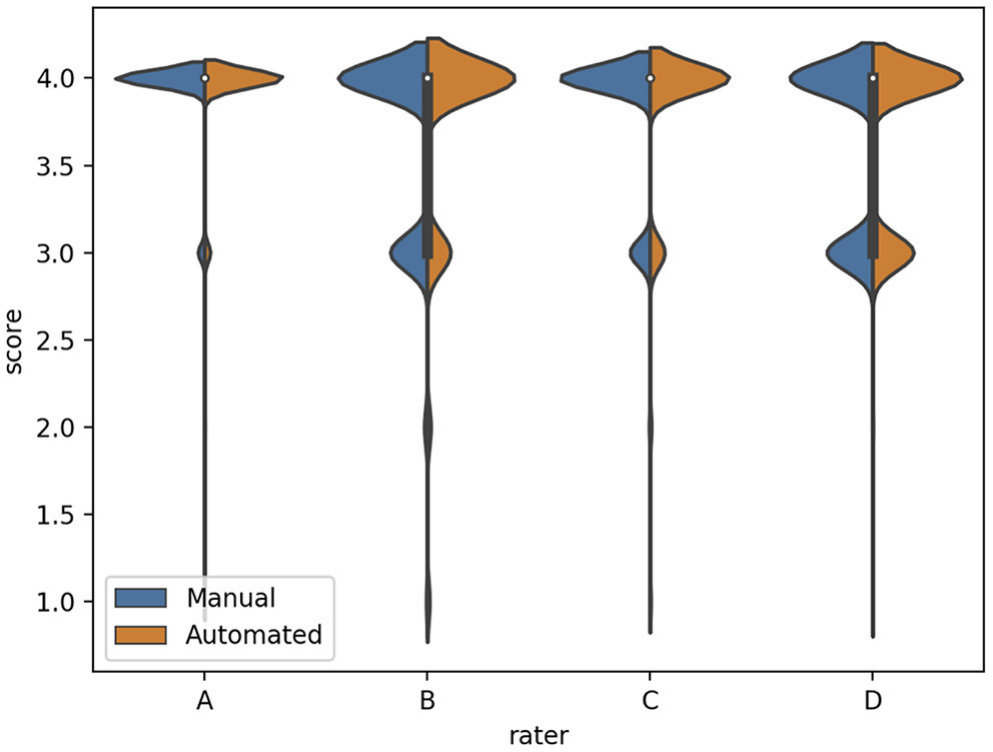
The distribution of quality scores for each rater for both sources of contours: manual (blue) and automated (orange) segmentation.

We subsequently investigated the distribution of the scores for each rater (Figure 3). We observed that the difference between the mean quality scores assigned to each segmentation method was not statistically significant for most evaluators, except for rater B (Table 1). The distribution of quality scores after excluding rater B, showed no significant difference between the two methods (OR (95% CI) = 1.05 (0.94–1.17); *p* = 0.40).

**Table 1:** Comparison of the mean quality score for manual and automated contours, and their corresponding Wilcoxon test p-value for statistical significance, for each rater.

**Rater**	**Mean quality score**		***P* value (Wilcoxon test)**
	**Manual**	**Automated**	
**A**	3.94	3.93	0.29 (*n* = 3,283)
**B**	3.67	3.71	<0.001 (*n* = 2,266)
**C**	3.81	3.81	0.87 (*n* = 3,281)
**D**	3.68	3.69	0.56 (*n* = 571)

The inter-observer agreement for manual and automated contours was also investigated using Gwet's AC2 agreement coefficient with ordinal weighting.

Overall, there was substantial inter-observer reliability for quality scoring of both manual and automated contours (AC2 = 0.937, 95% CI, (0.935–0.939)). In particular, the score agreement between all raters for automated contours was significantly better than for the manual segmentation (AC2 = 0.940, 95% CI, (0.937–0.943) and AC2 = 0.934, 95% CI, (0.931–0.937), respectively, *p* = 0.006).

**Figure 4:**
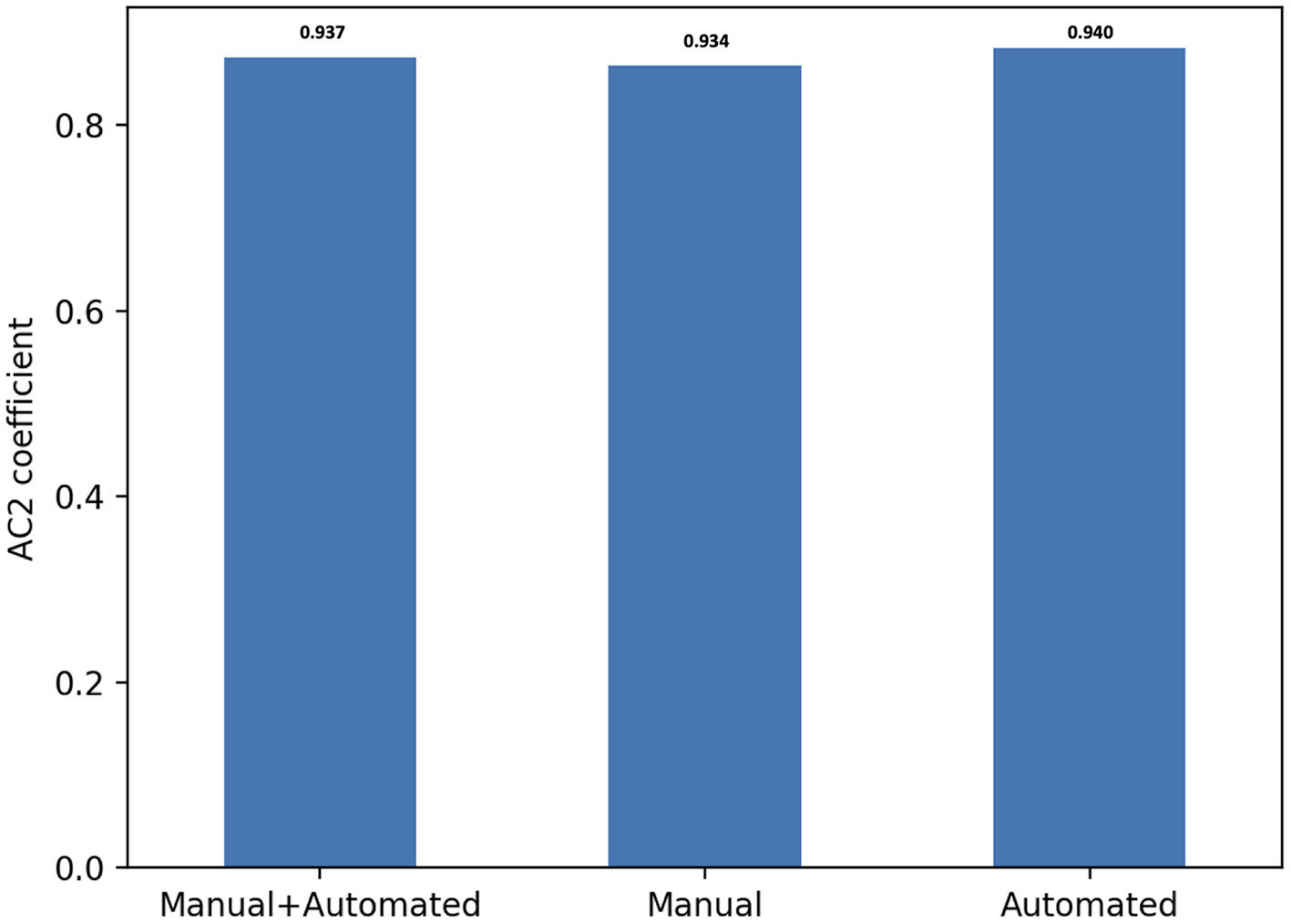
Score agreement between all raters for manual, automated contours and both segmentation methods. The interobserver reliability is expressed using Gwet's second-order agreement coefficient with ordinal weighting applied (AC2) (y axis).

All AC2 values were qualified as very good using the probabilistic benchmark procedure, with 100% membership probability for the interval 0.8–1.0. The inter-observer agreement for each method of contouring is shown in Figure 4.

### Quality Score Analysis According to the Contour Type, Slice Level and Underlying Disease

Delineating cardiac contours might be a challenging task in some circumstances, and this might negatively impact the clinical acceptability of segmentation. For instance, manual corrections might be needed when contouring areas with increased LV trabeculation, for apical slices or RV walls. Thus, to gain more insight into the variation in quality for both sources of contours, the scores were dichotomized using multiple factors.

**Figure 5:**
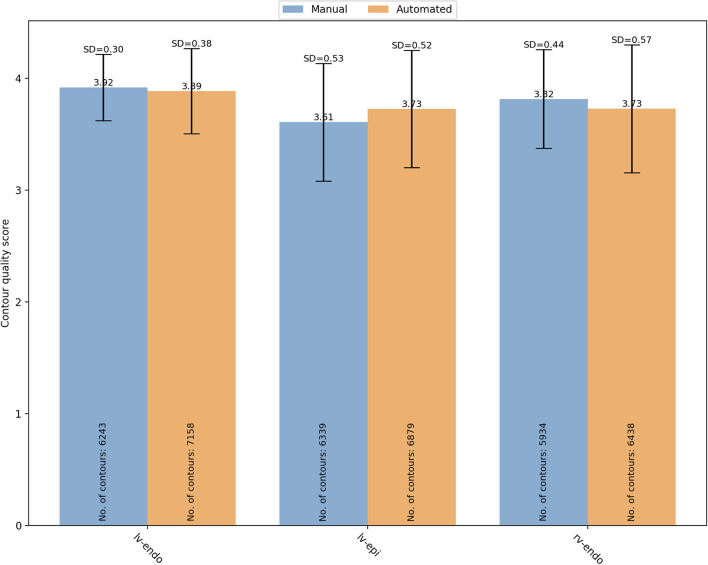
Overall mean quality scores for LV endocardial, LV epicardial and RV endocardial contours obtained from manual (blue) and automated (orange) segmentation. LV, left ventricle; RV, right ventricle; SD, standard deviation.

Firstly, we analyzed the distribution of quality scores between the two segmentation methods based on the contour type (Figure 5).

For manual segmentation, the mean quality scores by contour type were 3.92 ± 0.30 (*n* = 6,243), 3.61 ± 0.53 (*n* = 6,339), and 3.82 ± 0.44 (*n* = 5,934) for LV endocardial, LV epicardial, and RV endocardial contours, respectively. Compared to LV epicardial, both LV endocardial (OR (95% CI) = 22.89 (17.26–30.35) and RV endocardial (OR (95% CI) = 6.00 (4.74–7.58) had significantly higher quality (*p* < 0.0001). For automated segmentation, instead, the mean quality scores were 3.89 ± 0.38 (*n* = 7,158), 3.73 ± 0.52 (*n* = 6,879), and 3.73 ± 0.57 (*n* = 6,438) for LV endocardial, LV epicardial, and RV endocardial contours, respectively. Compared to LV epicardial, both LV endocardial (OR (95% CI) = 9.84 (7.09–13.66) and RV endocardial (OR (95% CI) = 1.68 (1.27–2.22) had significantly higher quality (*p* < 0.0001). A significant interaction was found suggesting that the differences in quality between contour types was more pronounced for the manual scores.

**Figure 6:**
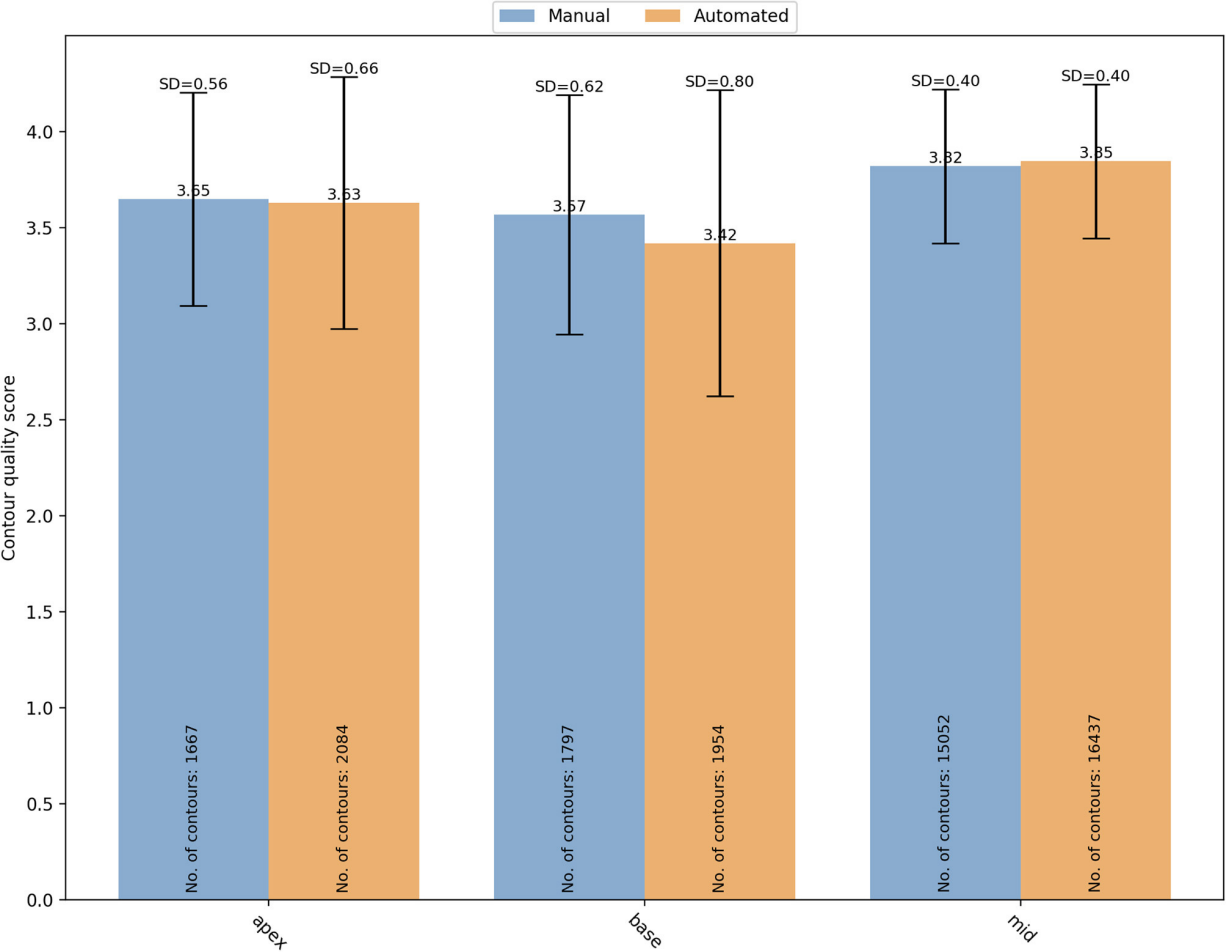
Distribution of the overall mean quality scores for different SAX slice levels (apical, basal and mid) with manual (blue) and automated (orange) segmentation. SD, standard deviation.

The segmentation complexity depends also on the slice level of the image. For instance, basal and apical images might be more challenging to contour than the mid ventricular images. For that reason, the contours quality scores were also analyzed based on the SAX slice level (Figure 6). For manual segmentation, the mean quality scores were 3.65 ± 0.56 (*n* = 1,667), 3.57 ± 0.62 (*n* = 1,797), and 3.82 ± 0.40 (*n* = 15,052), for apical, basal, and mid-ventricular levels, respectively. Compared to mid level, both basal (OR (95% CI) = 0.18 (0.13–0.25) and apical (OR (95% CI) = 0.44 (0.31–0.63) had significantly lower quality scores (*p* < 0.0001). Whereas the quality scores for the automated contours were 3.63 ± 0.66 (*n* = 2,084), 3.42 ± 0.80 (*n* = 1,954), and 3.85 ± 0.40 (*n* = 16,437), for apical, basal, and mid-ventricular levels, respectively. Compared to mid level, both basal (OR (95% CI) = 0.06 (0.04–0.09) and apical (OR (95% CI) = 0.29 (0.19–0.44) had significantly lower quality scores (*p* < 0.0001). A significant interaction was found (*p* < 0.0001) suggesting that reductions in quality for basal levels were more pronounced for the automated scores. Instead, the interaction was not significant for the apical levels (*p* = 0.17).

**Figure 7:**
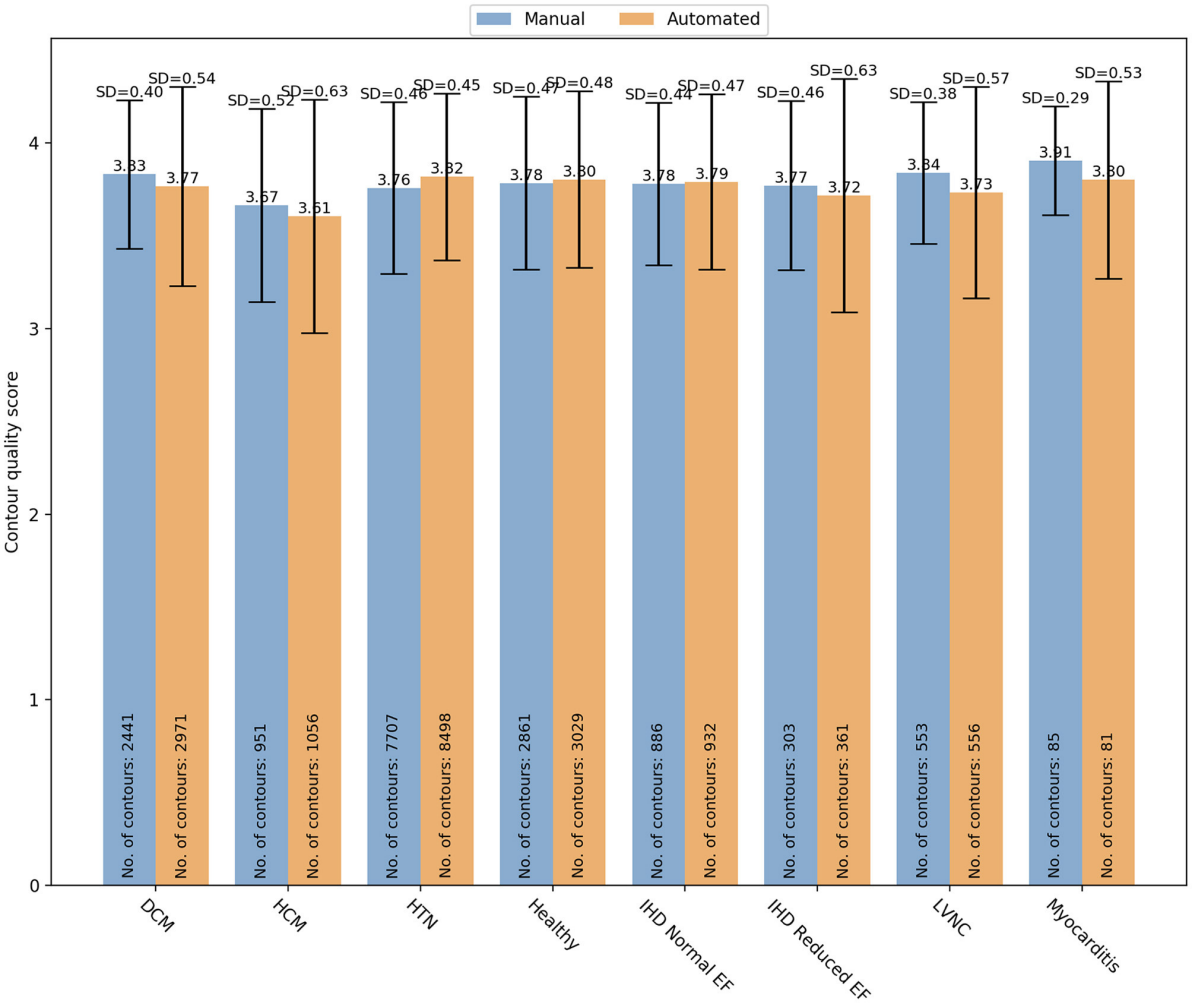
Distribution of the overall contour quality scores by different pathologies for manual (blue) and automated (orange) segmentation. DCM, dilated cardiomyopathy; HCM, hypertrophic cardiomyopathy; HTN, hypertension; IHD, ischaemic heart disease; EF, ejection fraction; LVNC, left ventricle non-compaction.

Finally, specific changes in cardiac structures associated with some conditions might also affect the contours quality. Therefore, we analyzed whether there was a variation in manual and automated quality scores based on the underlying pathology (Figure 7). In particular, we analyzed the quality score assigned for both manual and automated contours in the following subsets: DCM (manual: 3.83 ± 0.04 vs automated: 3.77 ± 0.54; OR (95% CI) = 0.59 (0.45–0.77); *p* < 0.0001), HCM (3.67 ± 0.52 vs 3.61 ± 0.63; OR(95% CI) = 0.83 (0.60–1.15); *p* = 0.27), HTN (3.76 ± 0.46 vs 3.82 ± 0.45; OR (95% CI) = 2.73 (2.22–3.37); *p* < 0.0001), healthy controls (3.78 ± 0.47 vs 3.80 ± 0.48; OR(95% CI) = 0.61 (0.36–1.00); p=0.051), IHD with normal (3.78 ± 0.44 vs 3.79 ± 0.47; OR(95% CI) = 1.44 (0.95–2.20); p=0.09) and reduced ejection fraction (EF) (3.77 ± 0.46 vs 3.72 ± 0.63; OR(95% CI_=0.72 (0.40–1.28); *p* = 0.26), LVNC (3.84 ± 0.38 vs 3.73 ± 0.57; OR(95% CI) = 0.67 (0.40–1.12); *p* = 0.13), myocarditis (3.91 ± 0.29 vs 3.80 ± 0.53; OR(95% CI)= 6.77 (0.70–65.43); *p* = 0.10). Only in DCM and HTN subgroups, there were significant differences in quality between the two contouring methods. In particular, manual contours received higher quality scores than the automated ones in DCM, while an opposite trend was observed for HTN.

## Discussion

Overall, our results showed significant clinical acceptability of automated contours based on quality scores assigned by four clinicians. Furthermore, there was substantial agreement between the evaluators in assessing the quality of both segmentation methods. In particular, the scoring agreement was significantly better for automated segmentation. These findings confirm the accuracy of this automated segmentation method based on its clinical acceptability.

Importantly, our QC approach allowed identification of scenarios where segmentation quality differed. Analysis of the quality scores by contour type and SAX slice level revealed a significantly lower quality of the LV epicardial contours, particularly with manual segmentation. Similar results were observed for the basal and apical contours. Furthermore, the reduction in quality for basal contours was more pronounced with the automated method. The scoring analysis by pathology, instead, did not show significant differences in contour quality between the two methods, except for DCM and HTN. Such patterns of lower segmentation quality would thus be subjected to further manual changes.

Therefore, our results on a small but varied image dataset confirm the ability of this method to rapidly assess the clinical acceptance of automated contours without using quantitative comparative metrics, which rely on the availability of hight quality, thoroughly validated “ground truth” data.

Furthermore, our scoring system has proven to be reliable among clinicians with different level of experience. Finally, we have shown that a systematic scoring analysis allows the identification of the poorly segmented images that may need further manual correction.

This study confirms the importance of a quality-based assessment of automated segmentation that directly relates to the clinical practice and might supplement other comparative metrics, as described below.

In most comparative studies, the automated segmentation performance is typically assessed on labeled validation datasets, distinct from the datasets used to train the ML algorithm. The most common evaluation approach is based on comparing the predicted segmentation against a “ground truth” reference, usually represented by manual expert annotations.

Several quantitative parameters can be used to measure the agreement between manual and automated segmentation, each one presenting different properties and suitability for specific tasks (12). The most used metrics are those based on position, surfaces distance, and volumetric overlap. In particular, the Dice similarity coefficient (DSC) and the Hausdorff distance (HD) are considered the best measures for the geometric quantification of boundary similarities. The DSC is a common measure of region overlap and it is mostly used in validating volume segmentations and measuring repeatability (13, 22). The HD, instead, is a distance-based measure that take into consideration the spatial position of voxels or distance between contours (12). These properties make the HD more suitable to validate algorithm where the boundary of segmented region is important (23).

Although these quantitative measures capture the differences between manual and automated contours, they might not necessarily predict whether they are clinically plausible or not.

A qualitative analysis focused on the clinical utility of contours is therefore essential to ensure the reliability of the automatically computed results and thus their acceptability in the clinical workflow.

The most common way to assess the quality of segmentation is based on expert manual inspection. However, besides being strongly subjective, visual QC is a time-consuming task, and it might be unfeasible to perform on large datasets.

Automated QC techniques can address these issues, and promising results have been already described in the literature (24, 25).

Despite some advantages over visual QC process, most of these methods still require large and fully annotated training datasets to evaluate the ML models. However, this might be challenging in real life, as we can mostly rely on relatively small sets of accurately segmented reference images.

Several ML and deep learning methods that do not require fully annotated datasets have been used to evaluate automated segmentation and estimate quantitative metrics (26, 27).

For instance, Robinson and colleagues used reverse classification accuracy (RCA) to predict the per-case quality of automated segmentations using only a small set of reference images and contouring (28). The RCA method of predicting quality of ML outputs is based on the assumption that a test case that happens to match the distribution of reference training images will result in high quality prediction while out of distribution (OOD) test cases will result in low quality predictions. With this assumption, a classifier is trained on both in-distribution and OOD data to predict the performance of the segmentation algorithm. However, such classifier when used as a discriminator alongside a segmentation network (generator) can improve segmentation quality on OOD cases, which is a basis of generative adversarial networks (GANs). By coupling a generator with a descriminator, the segmentation head of the GAN could then outperform the RCA classifier. In short, if a classification network is trained to accurately predict the performance of a segmentation network – as was done with the RCA approach – it implies that the segmentation algorithm may not have been sufficiently trained or was trained on a limited dataset (compared to the classification network). This approach also does not guarantee the generalizability of the classification network to other OOD test cases that the RCA was not trained on, thus limiting its utility to yet another subset of data.

Unlike Robinson's method, our approach aims to assess the clinical acceptability of automatic contours rather than predicting the segmentation accuracy. Furthermore, our systematic scoring analysis enables identifying cases where the segmentation has failed based on clinicians' judgment. Only these selected images would thus require further manual operator corrections. This will save time in the review process. Besides, clinical assessment of automated contours can potentially be obtained more efficiently across multiple institutions using our contours quality scoring system.

Furthermore, unlike other QC methods described so far, our approach captures the clinical acceptance of automated segmentation in a blinded manner. The observer clinicians do not know whether the evaluated contours are manual or automated by the algorithm when scoring them. Indeed, knowing the source of segmentation could influence their clinical decision and potentially introduce bias. Having blinded observers can thus minimize the risk of such unintended bias in the qualitative assessment.

A blind manner approach to evaluate the quality of automated contouring was also used by Gooding et al. who proposed a framework based on the Turing Test method, also referred to as the “Imitation Game” (29). This approach assumes that the inability of an interrogator to distinguish the machine behavior from the human behavior may indicate a good machine performance (30). Based on these assumptions, the authors presented contours from different thoracic organs to eight blinded clinical observers, who were asked to determine whether they were automated or manually generated. The inability of observers to correctly identify the source of contours was considered an indicator that the predicted contours were acceptable or at least of the same quality as the human standard, indicating a reduced need for manual editing. Furthermore, they found that the misclassification rate better predicted the time saved for editing auto-contours than other standard quantitative metrics, such as the DSC value.

Although the Turing indistinguishability might be considered a surrogate measure of automated contouring performance (31), it does not necessarily predict their clinical acceptability. Our approach, instead, is based on the assumption that if automated contours are judged of good quality by blinded observer clinicians, then they might be considered clinically acceptable. Therefore, our QC framework allows an evaluation of automated method more relevant to clinical applications and independent from how much the ML algorithm can imitate human performance.

Finally, since our method of assessing automated segmentation is not based on extensive manual expert annotations but on contour quality scores easily obtainable from clinicians, it might be used for quality controlling large-scale imaging datasets such as the UKB Imaging Study.

### Limitations

The dataset used for the scoring analysis was composed exclusively of good quality images. Therefore, although our method can be applied to real-life datasets with a similar level of image quality, care must be taken when generalizing such results to low-quality image datasets.

The manual segmentation and the quality assessment of all contours were performed by two different teams of clinicians, whose judgment was based on two different SOPs for post-processing analysis. Although this would account for some differences in the image interpretation, the scoring analysis by underlying pathology showed good agreement between the quality of automatic and manual contours in most cases. This suggests that the quality assessment was based on the clinical acceptability rather than the accuracy of the contour drawing and is more in line with a real-world clincal scenario. This indicates that our approach is suitable for QC analysis involving multiple centers with different institutional guidelines and clinicians with varying contour styles.

Although we did not assess the inter-observer variability between the NHCS operators for this specific study, an excellent inter-operator reproducibility of cardiac measurements was reported in a previous publication (14).

We did not evaluate the automated segmentation accuracy using quantitative metrics. Therefore, we could not study whether there was a correlation between quantitative metrics and clinical contour quality. However, that was beyond the scope of this paper. Our purpose was to present a method allowing a more clinically relevant evaluation of automated contouring, thus not based on the quantitative assessment of the ML algorithm.

We could not assess the intra-rater agreement across multiple instances in this study. However, the validated UKB standardized protocol used as a reference to grade contouring has produced good to excellent intra and inter-observer variability, as shown in a previous publication (18).

Finally, we did not assess whether factors like sex, age and patients' ethnicity could influence the contouring quality as data were fully anonymised. Future studies, perhaps on larger datasets integrating clinical and demographic information, might address this question.

## Conclusion

There is a growing need for a quality-based evaluation of automated segmentation more relevant to clinical practice and supplementing other quantitative measures of ML performance. Our systematic scoring analysis allows assessing automated contouring based on their clinical acceptability by involving clinicians directly during the algorithm validation process. This approach focused on the contours clinical utility could ultimately improve clinicians' confidence in AI and its acceptability in the clinical workflow.

## Data Availability Statement

The raw data supporting the conclusions of this article will be made available by the authors, without undue reservation.

## Ethics Statement

The studies involving human participants were reviewed and approved by National Health Center Singapore (NHCS). The patients/participants provided their written informed consent to participate in this study.

## Author Contributions

SEP, MO, AA-K, AS, MYK, and JMP conceived and designed the study. MO, JC, and ER performed data analysis. ER wrote the manuscript. T-TL, SAC, DJH, BA, D-FT, JB, and CWLC performed manual contours. The automated segmentation was performed by Circle Cardiovascular Imaging Inc. ER, SEP, MYK, and KF assessed the quality of both manual and automated contours. All authors read and approved the final manuscript.

## Funding

SEP acknowledges support from the National Institute for Health Research (NIHR) Biomedical Research Center at Barts. SEP acknowledges the British Heart Foundation for funding the manual analysis to create a cardiovascular magnetic resonance imaging reference standard for the UK Biobank imaging resource in 5000 CMR scans (www.bhf.org.uk; PG/14/89/31194). SEP acknowledges support from the SmartHeart EPSRC programme grant (www.nihr.ac.uk; EP/P001009/1). SEP and ER also acknowledge support from the London Medical Imaging and Artificial Intelligence Center for Value Based Healthcare (AI4VBH), which is funded from the Data to Early Diagnosis and Precision Medicine strand of the government's Industrial Strategy Challenge Fund, managed and delivered by Innovate UK on behalf of UK Research and Innovation (UKRI). N.A. recognizes the National Institute for Health Research (NIHR) Integrated Academic Training programme which supports his Academic Clinical Lectureship post. DJH is supported by the Duke-NUS Signature Research Programme funded by the Ministry of Health, Singapore Ministry of Health's National Medical Research Council under its Clinician Scientist-Senior Investigator scheme (NMRC/CSA-SI/0011/2017), Centre Grant (CGAug16M006), and Collaborative Centre Grant scheme (NMRC/CGAug16C006).

## Author Disclaimer

The views expressed are those of the authors and not necessarily those of the AI4VBH Consortium members, the NHS, Innovate UK, or UKRI.

## Conflict of Interest

SEP provides consultancy to and owns stock of Cardiovascular Imaging Inc, Calgary, Alberta, Canada. The remaining authors declare that the research was conducted in the absence of any commercial or financial relationships that could be construed as a potential conflict of interest.

## Publisher's Note

All claims expressed in this article are solely those of the authors and do not necessarily represent those of their affiliated organizations, or those of the publisher, the editors and the reviewers. Any product that may be evaluated in this article, or claim that may be made by its manufacturer, is not guaranteed or endorsed by the publisher.

## Abbreviations

AC2: second-order agreement coefficient
CI: confidence interval
CMR: cardiovascular magnetic resonance
CNN: convolutional neural network
CVDs: cardiovascular diseases
DCM: dilated cardiomyopathy
DCS: dice similarity coefficient
ED: end-diastole
ES: end-systole
GUI: graphical user interface
HD: hausdorff distance
HTN: hypertension
HCM: hypertrophic cardiomyopathy
IHD: ischaemic heart disease
LV: left ventricle
LVNC: left ventricular non-compaction
ML: machine learning
NHCS: national health center singapore
OR: odds ratios
QC: quality control
RCA: reverse classification accuracy
RV: right ventricle
SAX: short-axis
SOP: standard operating procedures
UKB: united Kingdom Biobank.
