# Supplementary material for: A Systematic Quality Scoring Analysis to Assess Automated Cardiovascular Magnetic Resonance Segmentation Algorithms
Source: Front Cardiovasc Med. 2022 Feb 15;8:816985. doi: 10.3389/fcvm.2021.816985 (PMC8886212; doi:10.3389/fcvm.2021.816985)
Supplement: Supplementary file 1 [file Table_1.pdf]

Supplementary methods

AC2 has been calculated as described by Gwet in a previous publication (1) using the formula below. Further explanation on the formula used can be found online (https://www.real-statistics.com/reliability/interrater-reliability/gwets-ac2/gwets-ac2-basic-concepts/).

$$AC2 = \frac{p_a - p_e}{1 - p_e}$$

r_i = the number of raters that assigned a rating to image i.

r_{ik} = the number of raters who assigned the kth score to image i.

n = the number of subjects which are rated by at least one rater;

n^* = the number of subjects which are rated by at least two raters;

q= the number of available ratings

w_{hk} are the weights in the Weights Table (supplementary table 1).

The formulas for p_a and p_e are defined as follows:

$$r_i = \sum_{k=1}^{q} r_{ik}$$

$$r_{ik}^* = \sum_{h=1}^{q} w_{kh} r_{ih}$$

$$p_i = \frac{1}{r_i(r_i - 1)} \sum_{k=1}^{q} r_{ik}(r_{ik}^* - 1) \qquad p_a = \frac{1}{n^*} \sum_{i=1}^{n^*} p_i$$

$$\pi_k = \frac{1}{n} \sum_{i=1}^{n} \frac{r_{ik}}{r_i} \qquad u = \sum_{h=1}^{q} \sum_{k=1}^{q} w_{hk}$$

$$p_e = \frac{u}{q(q-1)} \sum_{k=1}^{q} \pi_k(1 - \pi_k)$$

Here, the p_i are only defined when $r_i \geq 2$.

Results that are closer to each other are given a higher weighting than those further apart. The ordinal weights used for 4 possible outcomes are given in the weights table below, so that results agreeing completely have a weight of 1, whereas results differing by one point have a weight of 0.833 and those differing by 2 points have a weight of 0.5.

Supplementary table 1: Ordinal weights for Gwet's AC associated with scores 1:4

	1	2	3	4
1	1.000	0.833	0.500	0.000
2	0.833	1.000	0.833	0.500
3	0.500	0.833	1.000	0.833
4	0.000	0.500	0.833	1.000

REFERENCES

1. Gwet LK. Handbook of inter-rater reliability: The definitive guide to measuring the extent of agreement among raters. Advanced Analytics, LLC, Gaithersburg, MD (2014).
